# 5.1 DIMENSIONS OF PSYCHOSIS AND THEIR TRAJECTORIES DURING TWO DECADES AFTER FIRST HOSPITALIZATION

**DOI:** 10.1093/schbul/sby014.013

**Published:** 2018-04-01

**Authors:** Roman Kotov

**Affiliations:** Stony Brook University

## Abstract

**Background:**

Heterogeneity of psychosis presents significant challenges for classification. Between two and 12 symptom dimensions have been proposed, and consensus is lacking. The present study sought to identify uniquely informative models by comparing the validity of these alternatives. A critical validator is future course, and we examined trajectory of each dimension.

**Methods:**

We investigated this question in the first U.S. study to follow an epidemiological cohort with psychotic disorders for 20 years after first hospitalization. Participants were assessed in person 6 times over 2 decades on Global Assessment of Functioning (GAF), psychotic symptoms, and mood symptoms, and 373 completed 20-year follow-up (68% of survivors) including an electrophysiological assessment of error processing. We first analyzed a comprehensive set of 49 symptoms rated by interviewers at baseline, progressively extracting from one to 12 factors. Next, we compared the ability of resulting factor solutions to (a) account for concurrent neural dysfunction and (b) predict 20-year role, social, residential, and global functioning, and life satisfaction.

**Results:**

A four-factor model showed incremental validity with all outcomes, and more complex models did not improve explanatory power. The four dimensions—reality distortion, disorganization, inexpressivity, and apathy/asociality—were replicable in 5 follow-ups, internally consistent, stable across assessments, and showed strong discriminant validity. On all of these measures schizophrenia exhibited a decline that began between years 5 and 10. Correspondingly, GAF scores dropped from 49 (Year 4) to 36 (Year 20). Neither aging nor changes in antipsychotic treatment accounted for the declines.

**Discussion:**

These results reaffirm the value of separating disorganization and reality distortion, are consistent with recent findings distinguishing inexpressivity and apathy/asociality, and suggest that these four dimensions are fundamental to understanding neural abnormalities and long-term outcomes in psychosis. They also revealed a substantial symptom burden across psychotic disorders that increased with time and ultimately may undo initial treatment gains. Additional research is needed, but previous studies suggest sociocultural factors and different care models may preempt this decline.

